# Prenatal SARS-CoV-2 infection results in neurodevelopmental and behavioral outcomes in mice

**DOI:** 10.1172/jci.insight.179068

**Published:** 2024-05-23

**Authors:** Courtney L. McMahon, Erin M. Hurley, Aranis Muniz Perez, Manuel Estrada, Daniel J. Lodge, Jenny Hsieh

**Affiliations:** 1 Department of Neuroscience, Developmental and Regenerative Biology, and; 2 Brain Health Consortium, University of Texas at San Antonio, San Antonio, Texas, USA.; 3 Department of Pharmacology and Center for Biomedical Neuroscience, University of Texas Health Science Center, San Antonio, Texas, USA.

**Keywords:** COVID-19, Neuroscience, Behavior, Neurodevelopment, Neurological disorders

## Abstract

Prenatal exposure to viral pathogens has been known to cause the development of neuropsychiatric disorders in adulthood. Furthermore, COVID-19 has been associated with a variety of neurological manifestations, raising the question of whether in utero SARS-CoV-2 exposure can affect neurodevelopment, resulting in long-lasting behavioral and cognitive deficits. Using a human ACE2–knock-in mouse model, we have previously shown that prenatal exposure to SARS-CoV-2 at later stages of development leads to fetal brain infection and gliosis in the hippocampus and cortex. In this study, we aimed to determine whether infection of the fetal brain results in long-term neuroanatomical alterations of the cortex and hippocampus or in any cognitive deficits in adulthood. Here, we show that infected mice developed slower and weighed less in adulthood. We also found altered hippocampal and amygdala volume and aberrant newborn neuron morphology in the hippocampus of adult mice infected in utero. Furthermore, we observed sex-dependent alterations in anxiety-like behavior and locomotion, as well as hippocampal-dependent spatial memory. Taken together, our study reveals long-lasting neurological and cognitive changes as a result of prenatal SARS-CoV-2 infection, identifying a window for early intervention and highlighting the importance of immunization and antiviral intervention in pregnant women.

## Abstract 2

SARS-CoV-2 exposure in utero causes developmental and neurological deficits in mice, including reduced size, increased anxiety-like behavior, decreased spatial memory, and aberrant neural architecture.

## Graphical abstract

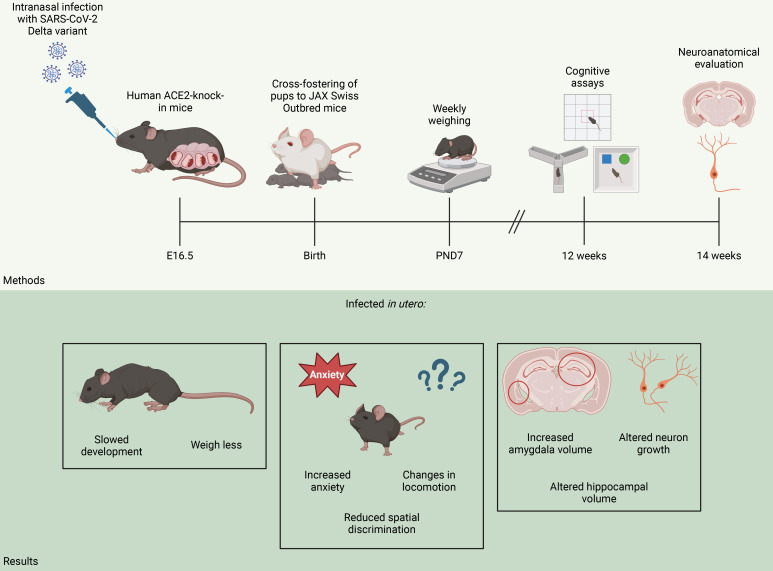

## Introduction

Growing evidence indicates that aberrant fetal brain development resulting from prenatal exposures, such as infection of the CNS by SARS-CoV-2 and the subsequent maternal and fetal immune responses, can lead to neurological deficits that persist into adulthood (1–11). Resulting behavioral consequences of prenatal viral infections include the development of autism spectrum disorder (ASD), schizophrenia, bipolar disorder, and anxiety and depressive disorders, among others, but may also contribute to the development of neurodegenerative disorders, such as Alzheimer’s disease, much later in life (4, 10, 12–24). Interestingly, males tend to be more susceptible to in utero infection and the resulting neurological deficits (25–27). While neurodevelopmental alterations are well established following infection of viruses with no known neurotropism, neurological symptoms (23, 24, 28–32) and confirmed CNS infection (33–37) are common in patients with COVID-19, increasing the need for a better understanding of the consequences of fetal brain infection by SARS-CoV-2.

Currently, various pregnancy complications are associated with maternal COVID-19 infection during pregnancy such as preterm birth, miscarriage, and placental damage. More recently, deficits have been observed in infants, including motor and social skill impairments, seizures, and acquired microcephaly (38–40). Furthermore, studies have found detectable levels of the SARS-CoV-2 spike protein in the human fetal brain, placenta, and other tissues (41–45). Our previous study using human ACE2–knock-in (hACE2-KI) mice found that vertical transmission of SARS-CoV-2 occurred, that the risk of infection of the fetal brain was highest when maternal infection occurred during the second or third trimesters, and that most cell types of the CNS were susceptible to infection (26). We also observed significant levels of gliosis in the cortex and hippocampus of the fetal brains. Importantly, our mouse model corroborated clinical studies of maternal COVID-19 infection, such as increased severity of infection during pregnancy and a higher incidence of preterm birth (46–49), demonstrating its reliability as a model for prenatal COVID-19 infection.

Here, we investigated the behavioral and neurological consequences of prenatal SARS-CoV-2 infection of the fetal mouse brain in order to predict human outcomes in the coming years. In this study, we found both developmental and behavioral differences in adult mice infected in utero compared with adult mock-infected mice, and we found increased amygdala volume, altered hippocampal morphology, and dysregulation of newborn neurons in the dentate gyrus of infected mice. This raises concerns regarding babies affected by maternal COVID-19 in utero. We hope that the results of our study will provide a better understanding of the neurological consequences and responsible mechanisms of prenatal SARS-CoV-2 brain infection, ultimately leading to early interventions and preventative strategies in at-risk children with direct CNS infection.

## Results

### Infected mice show slowed development and weigh less in adulthood.

**Figure 1:**
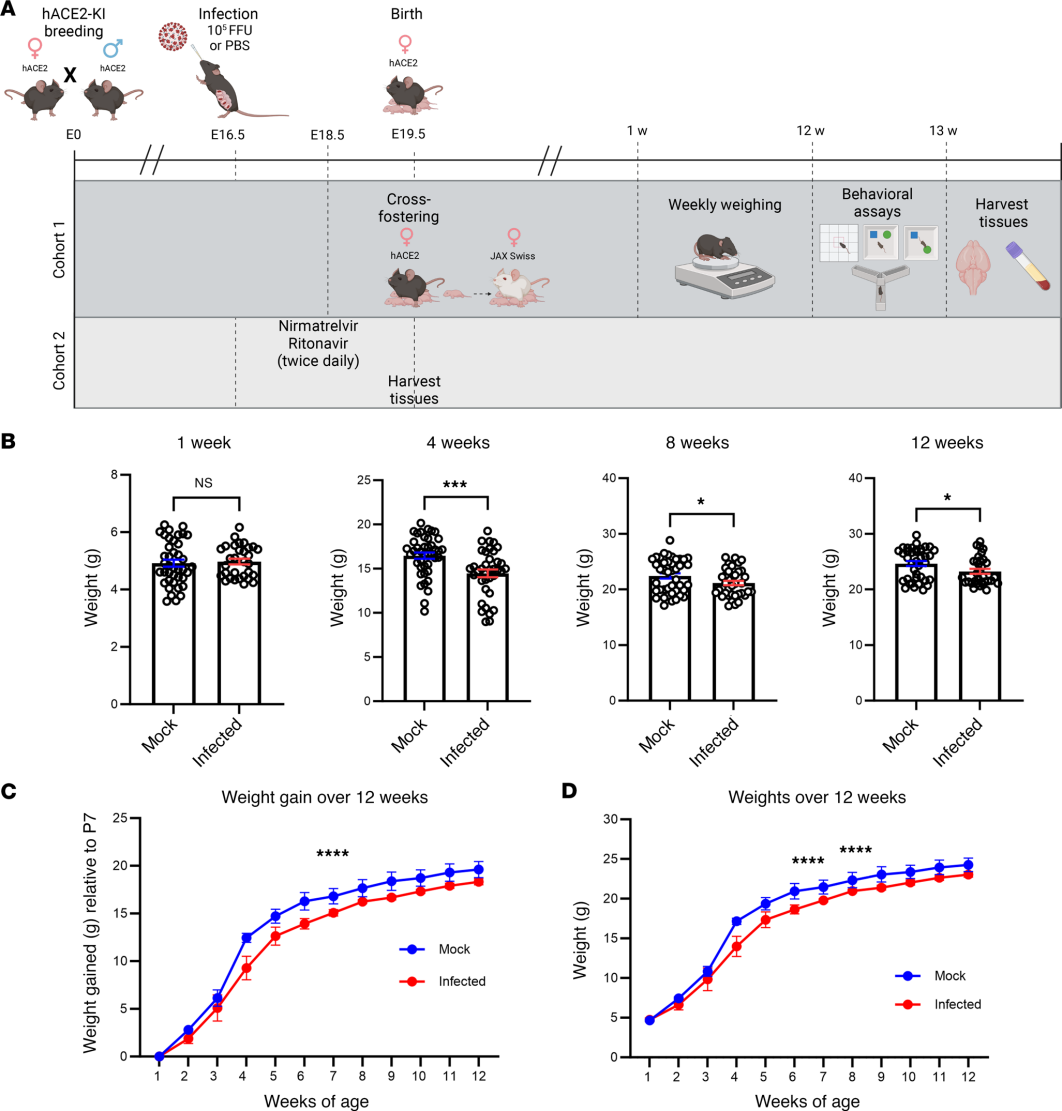
Developmental deficits are seen in mice infected in utero with SARS-CoV-2. (**A**) Experimental schematic. (**B**) Weights of mock-infected vs. infected mice by weeks of age. *n* = 30–40. (**C**) Infected mice gain weight slower than mock-infected mice. (**D**) Infected mice remain smaller in adulthood than mock-infected mice. *n* = averages of 5–6 litters per infection group. Student *t* tests and repeated measures 1-way ANOVA were used to evaluate statistical significance. *P < 0.05, ***P < 0.001, and ****P < 0.0001.

Homozygous hACE2-KI mice were bred and infected with 1 × 10^5^ FFU of SARS-CoV-2 at E16.5 (Figure 1A). Immediately after birth, all pups from both the mock-infected and infected groups were cross-fostered to JAX Swiss Outbred mice to account for aberrant maternal care following infection that could lead to developmental deficits in offspring. To confirm similar infection levels, the lungs of the hACE2-KI dams were harvested for quantitative PCR (qPCR) analysis (Supplemental Figure 1; supplemental material available online with this article; https://doi.org/10.1172/jci.insight.179068DS1). Starting at P7, all pups were weighed weekly on the same day until 12 weeks old. Our previous study showed that E18.5 fetuses and P1 pups infected in utero weighed more than those of the mock-infected group (26); however, we found in this study that infected pups began to weigh similarly to mock-infected pups by 1 week of age (Figure 1B), continued to gain weight slower (Figure 1C), and gained less weight overall (Figure 1, B and D) over the course of 12 weeks. Both male and female infected mice were, on average, 2–3 g less than mock-infected mice at maturity. The most significant weight differences were observed at week 4, which is typically a rapid period of development in mice following weaning (50), with a 5 g difference between mock-infected and infected mice.

### Infected mice display significant anxiety-like behavior and locomotion differences in adulthood.

**Figure 2:**
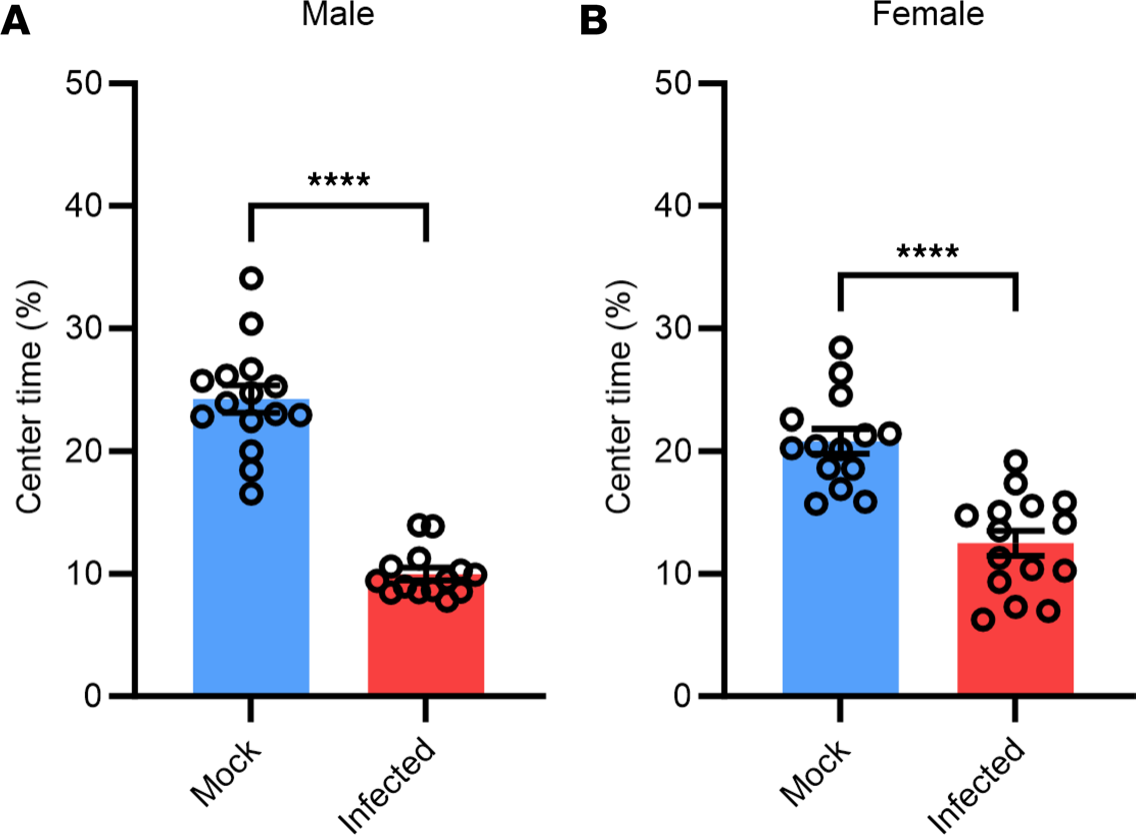
Infected mice display significant anxiety-like behavior compared with mock-infected mice. (**A**) Male mock-infected vs. infected mice. (**B**) Female mock-infected vs. infected mice. Significant outliers removed for analysis. *n* = 14–15. Student *t* tests were used to evaluate statistical significance. ****P < 0.0001.

Due to the presence of SARS-CoV-2 infection in the hippocampus and cortex of P1 pups infected at E16.5 (Supplemental Figure 2), we investigated whether or not behavioral consequences occurred in adulthood following prenatal infection. Anxiogenic behavior and locomotion were evaluated in both the prenatally mock-infected and infected mice at 12 weeks of age using the open field test (OFT). Briefly, an open arena was divided into a central zone and a peripheral zone, and the amount of time the mouse spent in each zone was recorded over a period of 10 minutes. Mice infected with SARS-CoV-2 in utero spent significantly less time in the center zone than the peripheral zone (10%–12%) compared with mock-infected mice (21%–23%) (Figure 2), suggesting an increased level of anxiety-like behavior. Because our previous study showed that male fetuses exposed in utero had higher levels and higher rates of infection than females (26), we evaluated anxiogenic behavior by sex. We found no difference between the sexes in this assay.

**Figure 3:**
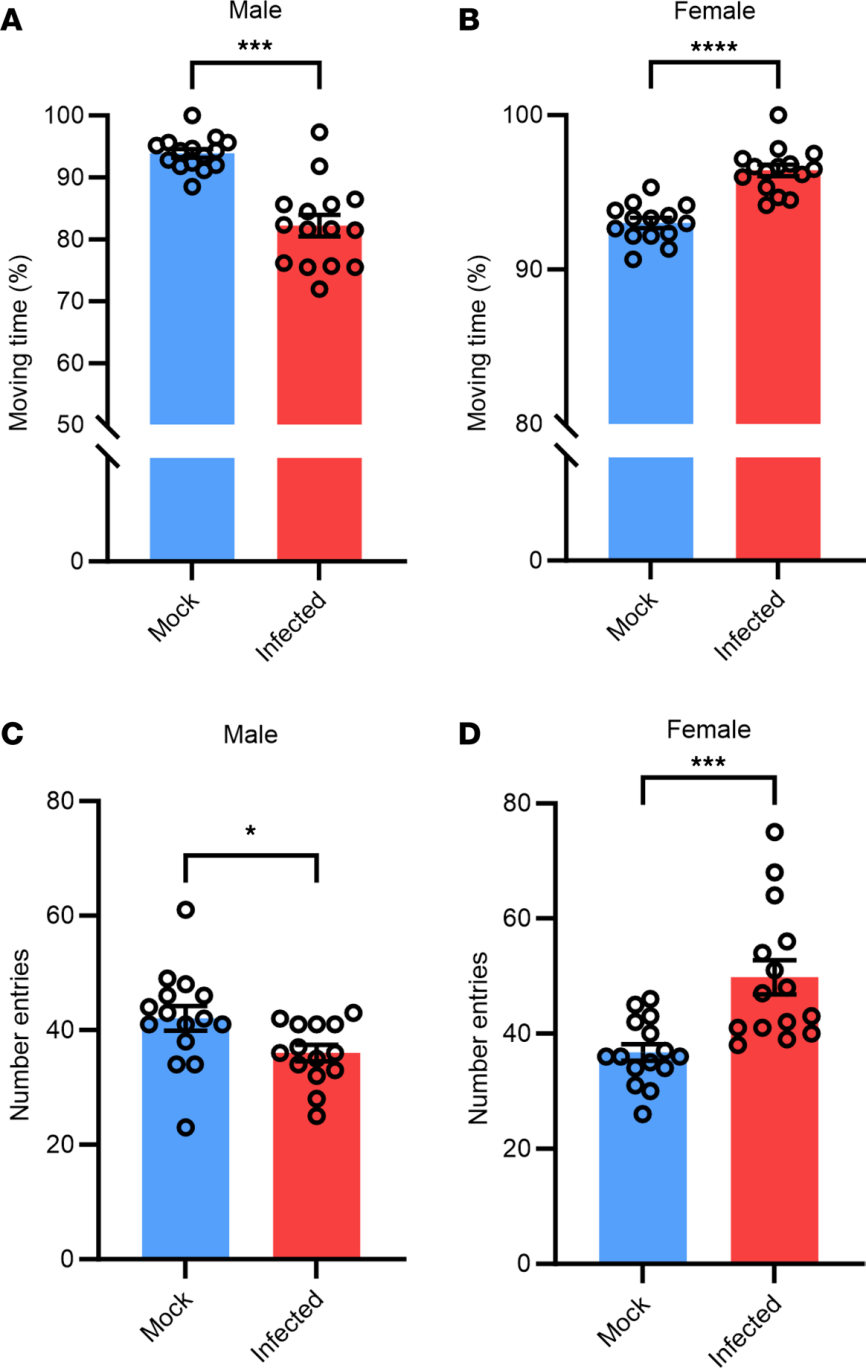
Infected mice display locomotion differences compared with mock-infected mice. (**A**) Male mock-infected vs. infected mice in OFT. (**B**) Female mock-infected vs. infected mice in OFT. (**C**) Male mock-infected vs. infected mice in Y-maze. (**D**) Female mock infected vs. infected mice in Y-maze. Significant outliers removed for analysis. *n* = 14–15. Student *t* tests were used to evaluate statistical significance. *P < 0.05, ***P < 0.001, and ****P < 0.0001.

We then evaluated changes in locomotion between mock-infected and infected mice. Due to the restrictions of performing these studies in an animal Biosafety Level 3 (ABSL3), we first evaluated locomotion by comparing the amount of time spent moving vs. time spent freezing in each 10-minute OFT session (Figure 3, A and B). Interestingly, we found that infected males displayed decreased locomotion compared with those mock infected (~13% less), while infected females displayed increased locomotion compared with those mock infected (~5% more). These results were corroborated by the results seen in the Y-maze spontaneous alternation test, which showed a decreased number of arm entries in males (~7 less) (Figure 3C) and an increased number of arm entries in females (~12 more) (Figure 3D). Together, these behavioral data suggest neurodevelopmental alterations in the amygdala and prefrontal cortex (PFC) regions of the prenatally infected mice.

### Spatial discrimination is significantly reduced in infected mice.

**Figure 4:**
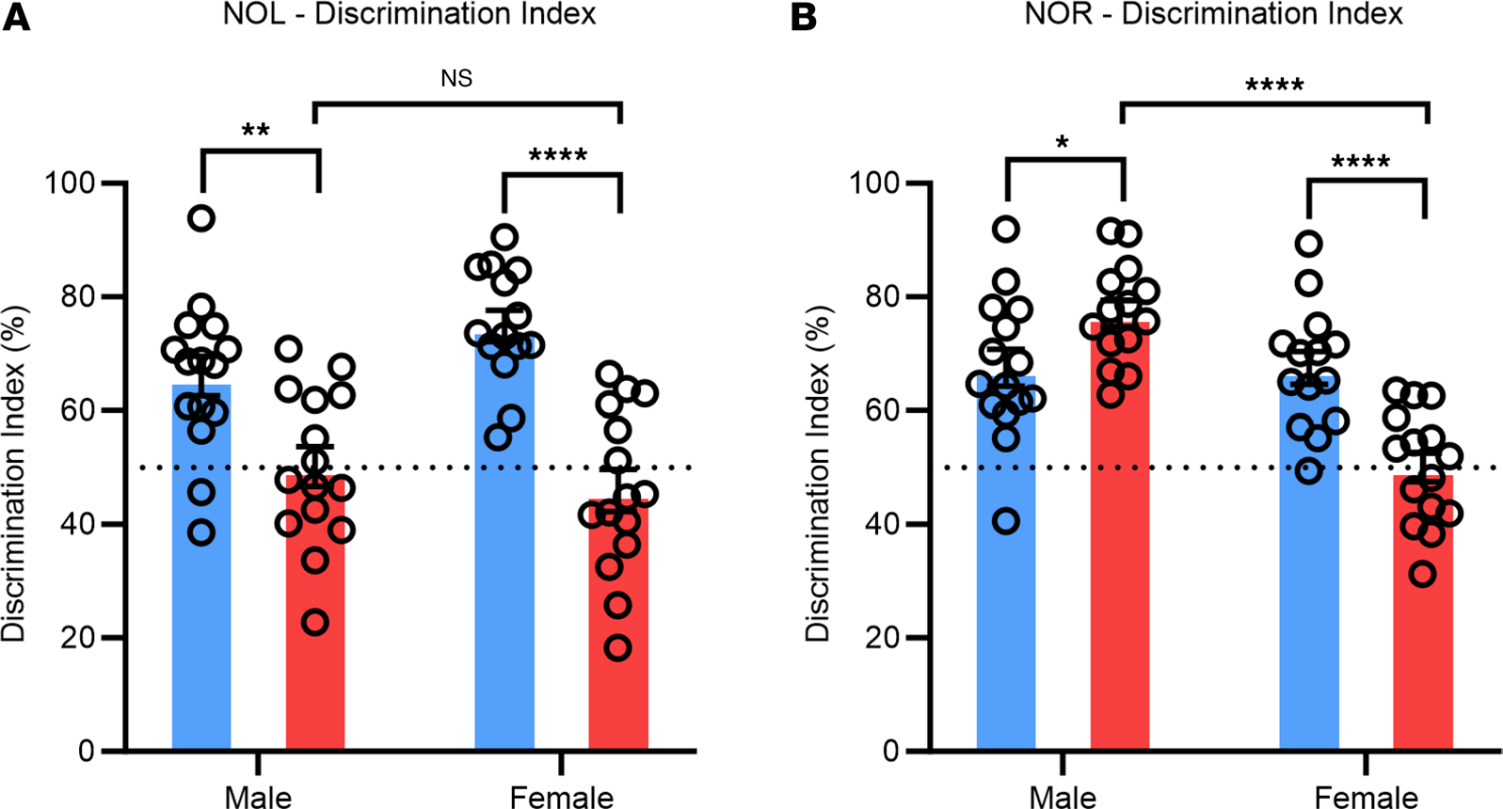
Spatial and novel object discrimination is seen in infected mice. (**A**) Infected mice display significantly reduced spatial discrimination. (**B**) Infected female mice display significantly reduced novel object discrimination. Significant outliers removed for analysis. *n* = 14–15. Repeated measures one-way ANOVA were used to evaluate statistical significance. *P < 0.05, **P < 0.01, ***P < 0.001, and ****P < 0.0001.

To investigate deficits in hippocampal function, the novel object location (NOL) test was used to evaluate spatial working memory. Mice were placed in the arena with 2 objects for 5 minutes for sampling, and they then returned to their home cage for a 5-minute delay before a testing session of 3 minutes (Supplemental Figure 3A). The time spent investigating both the familiar object and the novel (moved) object were recorded (Supplemental Figure 4), and the discrimination index was calculated. Infected mice displayed significantly decreased spatial discrimination with a discrimination index of approximately 50% compared with approximately 64%–75% in mock-infected mice, suggesting a deficit in spatial working memory (Figure 4A). No differences were observed between the sexes in this assay.

The Y-maze spontaneous alternation assay was also used to investigate spatial working memory deficits. The sequence of arm entries for each mouse was recorded, and the percent alternation was calculated; however, no differences were seen between the infected and mock-infected mice in this assay (Supplemental Figure 5). These data may be indicative of less severe perturbations in the hippocampus of these mice.

### Female infected mice display reduced novel object discrimination, while the males do not.

The novel object recognition (NOR) test was used to further investigate neurological alterations in infected mice. The same sampling and delay conditions used in the NOL were used for the NOR test, with the exception of 1 object being replaced with a novel object rather than moving the object to a novel place (Supplemental Figure 3B). The time spent interacting with both the familiar and novel objects was recorded (Supplemental Figure 6), and the discrimination index was calculated using the same equation used for NOL. Interestingly, only female infected mice showed a decrease in novel object discrimination at approximately 50% compared with 67% in mock-infected mice (Figure 4B). Male infected mice displayed a statistically significant increase in object discrimination; however, no biological relevance is attributed to this difference.

### Seropositivity to SARS-CoV-2 NP persists after 14 weeks but is not correlated with behavioral phenotypes.

We next conducted ELISA to determine if seropositivity to the SARS-CoV-2 nucleocapsid protein (NP) persisted after 14 weeks and found that it did persist in approximately 50% of infected mice (Supplemental Figure 7A). We classified any values above the values recorded in mock-infected mice as seropositive. We found a positive response in 42% of males and in 61% of females at this time point (Supplemental Figure 8). To determine if persisting seropositivity was correlated with more severe behavioral phenotypes, we next compared these levels with behavioral scores; however, we found that there was no correlation between these values (Supplemental Figure 7B). We also conducted qPCR of the brains of the infected mice and found no persisting SARS-CoV-2 RNA at 14 weeks (data not shown).

### Amygdala and hippocampal volume are significantly altered in infected mice compared with mock-infected mice.

**Figure 5:**
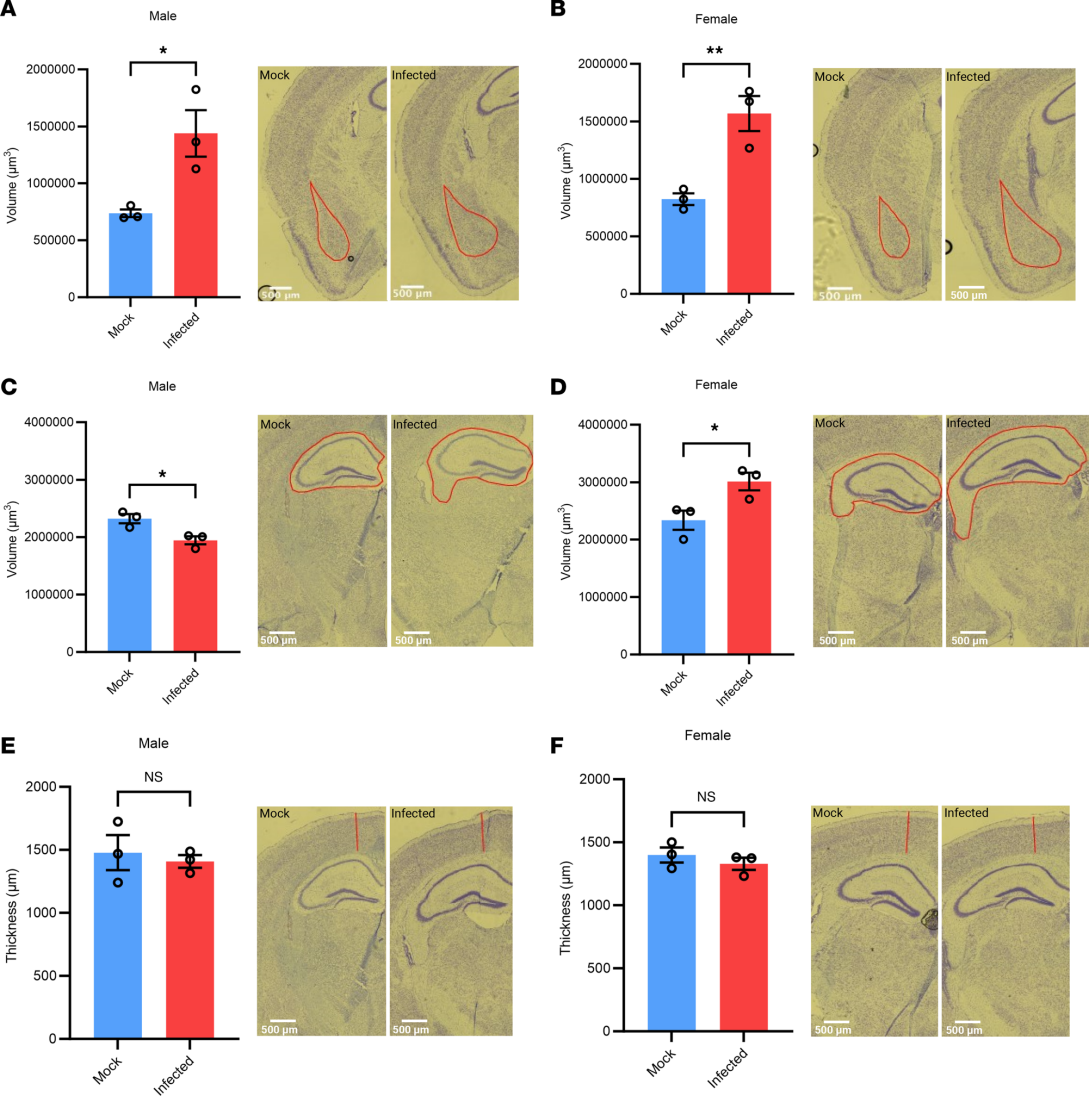
Amygdala and hippocampal volume are significantly altered in infected mice. Amygdala volume is significantly increased in both sexes (**A** and **B**), and hippocampal volume is significantly increased in females but decreased in males (**C** and **D**) in infected compared with mock-infected mice. No significant change is seen in cortical volume (**E** and **F**). Representative images of different regions of the same brain are shown. *n* = 3. Student *t* tests were used to evaluate statistical significance. *P < 0.05, **P < 0.01, ***P < 0.001, and ****P < 0.0001. Scale bars: 500 μm.

We next sought to identify any morphological differences in the brain regions underlying the behavioral changes seen in infected mice. We first determined if there were changes in amygdala volume, as this is the brain region most associated with anxiety-related behaviors (51). We found a significant increase in amygdala volume in both the male and female infected mice, with infected mice having double the volume as mock-infected mice (Figure 5, A and B). We next measured hippocampal volume, as the hippocampus is implicated in location memory, and found significant differences between infected and mock-infected mice in both the males and females (Figure 5, C and D). Interestingly, infected males had a decreased hippocampal volume of approximately 6% compared with mock-infected mice (Figure 5C), while infected females had an increased volume of approximately 4% compared with mock-infected mice (Figure 5D). Alternatively, we found no significant differences in cortical thickness between infected and mock-infected mice (Figure 5, E and F).

### Newborn neuron growth is altered in infected mice.

**Figure 6:**
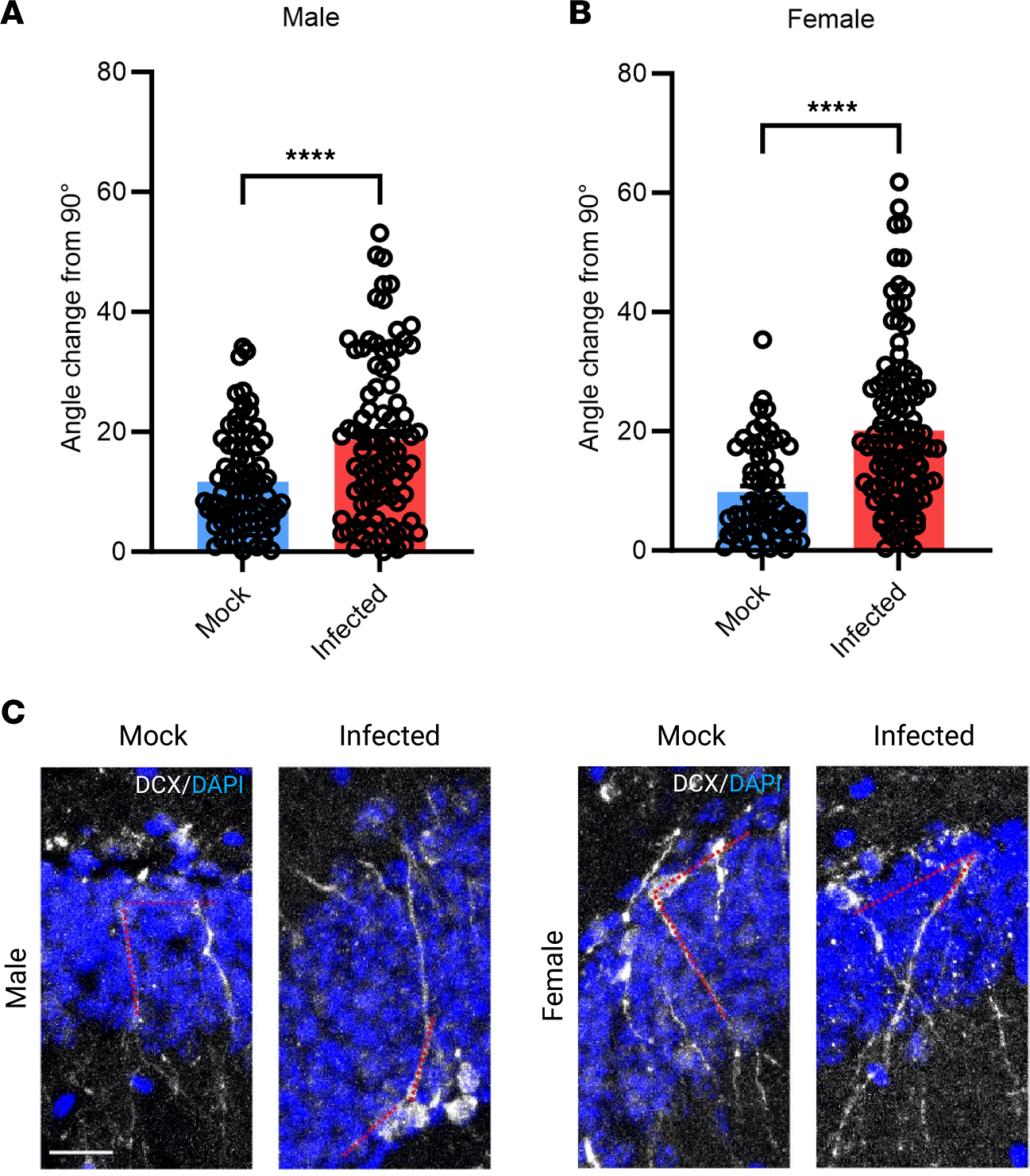
Newborn neuron growth is altered in infected mice. (**A** and **B**) DCX angle in DG is altered in infected mice compared with control. (**C**) Representative images. Scale bar: 200 μm (inset: 20 μm). *n* = 26–30 neurons/mouse from 3 mice. Student *t* tests were used to evaluate statistical significance. ****P < 0.0001.

We then investigated whether there were underlying cellular deficits within the hippocampus that could contribute to behavioral differences. Previous studies have shown aberrant neurogenesis following increased inflammatory processes, such as a reduction in newborn neurons and abnormal newborn neuron morphology (52, 53). We measured newborn neuron expression using doublecortin (DCX) in infected and mock-infected mice and found no significant differences (data not shown). Furthermore, as a surrogate measure of proper newborn neuron incorporation into the dentate gyrus, and because aberrant newborn neuron morphology is a feature of neuropathology in mouse models of seizures (54), we measured the dendritic angle compared with the subgranular zone. We found that, in both male and female infected mice, there was a deficit in proper growth direction of the newborn neurons (Figure 6). The dendritic angle should be close to 90°, since the newborn neurons should incorporate into the dentate gyrus perpendicularly, and while the average angle change in the mock-infected mice was ± 10°, the average angle change in infected mice was ± 20°, with many reaching a change of ± 30°. This suggests a continued aberrant neurogenesis process in adulthood.

### TREM2 expression is decreased in female infected mice.

Our previous studies found high levels of gliosis in the fetal brain following a SARS-CoV-2 infection (26). To determine if this continues in adult mice, we next evaluated the levels of GFAP and Iba1 expression in the brains at 14 weeks. We found no differences in GFAP or Iba1 levels in infected vs. mock-infected mice (data not shown). We then used IHC to evaluate the percentage of TREM2 fluorescence expression in the adult mice, which is a risk factor for neurodegenerative diseases and a regulator of microglial function in the brain (55). Surprisingly, we found a slight decrease in TREM2 levels in female infected mice compared with mock-infected mice but not in male infected mice (Supplemental Figure 9). This suggests a possibly higher risk of neurodegeneration in females later in life.

### Treatment with nirmatrelvir and ritonavir decreased infection in pregnant dams but did not prevent vertical transmission of SARS-CoV-2.

**Figure 7:**
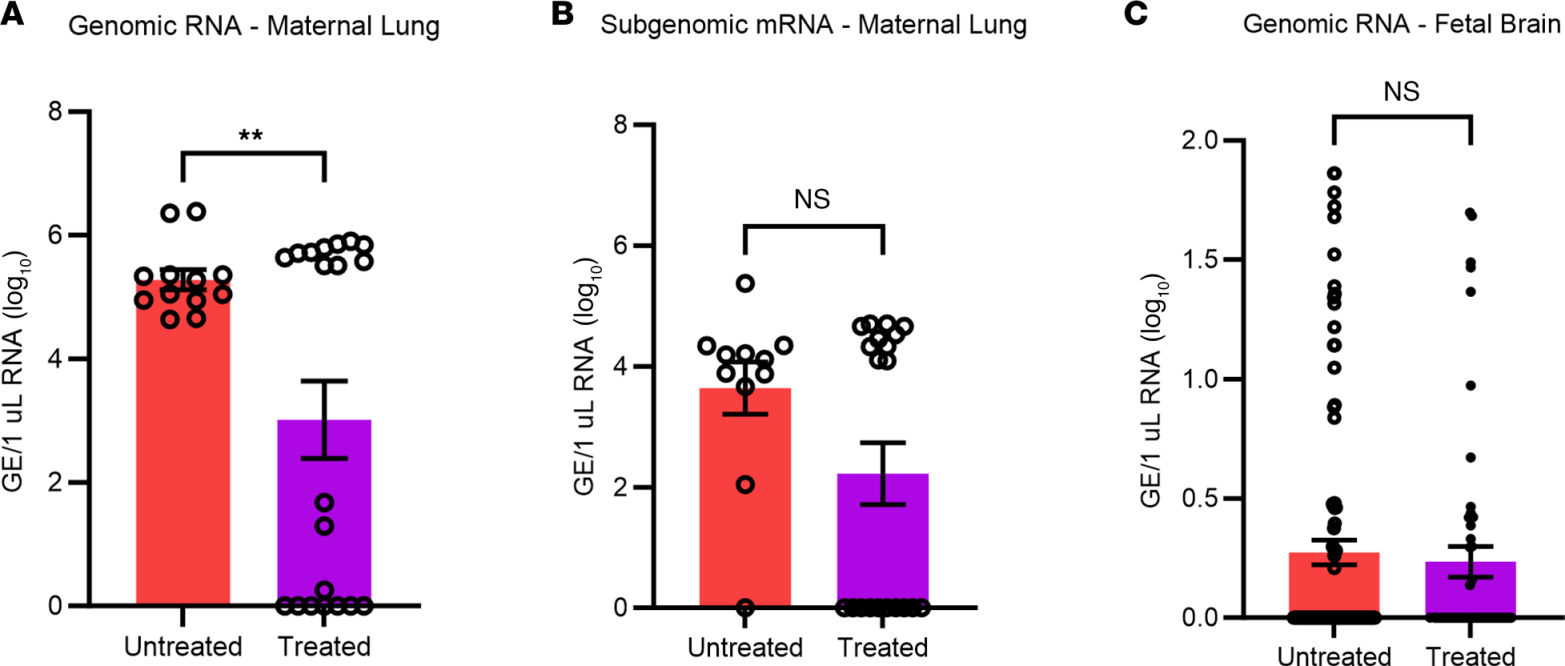
Treatment with nirmatrelvir and ritonavir decreased maternal SARS-CoV-2 infection but did not prevent viral transmission to fetus. (**A**) Genomic SARS-CoV-2 RNA detected in the maternal lung. (**B**) Subgenomic viral mRNA in the maternal lung. *n* = 7–15. (**C**) Genomic SARS-CoV-2 detection in the fetal brain. *n* = 50–60. Student *t* tests were used to evaluate statistical significance. **P < 0.01.

To investigate whether the currently used antiviral in severe or high-risk COVID-19 cases, Paxlovid (nirmatrelvir/ritonavir), could prevent viral transmission to the fetal brain, we treated our experimental group at E16.5 with 5 mg nirmatrelvir and 1.5 mg ritonavir every 12 hours starting at 24 hours postinfection (hpi). This dosage was used to replicate the clinical treatment regimen of 300 mg nirmatrelvir and 100 mg ritonavir/40 kg twice daily (56). In clinical cases, Paxlovid treatment continues for 5 days; however, we treated pregnant mice until E18.5, which we determined to be the time point of peak infection in our model (26). We then harvested the lungs of the dams and the fetal brains for qPCR analysis. We found that this antiviral treatment eliminated infection levels (Figure 7A) and viral replication levels (Figure 7B) in 50% of the pregnant infected dams compared with untreated controls. Additionally, in the dams that the antivirals were ineffective against, vertical transmission of SARS-CoV-2 to the fetal brain was not decreased (Figure 7C). No viral replication was detected in the fetal brain in either group (data not shown).

## Discussion

This study sought to investigate the potential neurodevelopmental and behavioral consequences of in utero exposure to SARS-CoV-2. Neurological symptoms have been seen in COVID-19 cases since early in the pandemic, and maternal exposure to viruses during pregnancy has been shown to cause neurological deficits in children later in life (10, 12, 14, 31, 57). These deficits may arise as a result of direct viral infection and damage of the fetal brain tissue or as a result of maternal immune activation (MIA). When MIA occurs, maternal inflammatory molecules can cross the placenta and the undeveloped blood-brain barrier of the fetus, resulting in neuroinflammation and neurodevelopmental deficits (58).

Studying the potential long-term effects of aberrant neurodevelopment in children following in utero exposure to SARS-CoV-2 is critical. Our previous study showed that vertical transmission of SARS-CoV-2 could occur during maternal COVID-19 infection, that the virus could infect the fetal brain, and that various cell types of the fetal brain were susceptible; however, maternal inflammation levels were low to undetectable in this study (26). This, in combination with the presence of SARS-CoV-2 RNA and proteins in the fetal brain tissue, suggests that direct viral infection rather than MIA is responsible for the neurological effects seen in this study. Here, we infected E16.5 pregnant mice with 1 × 10^5^ FFU of SARS-CoV-2, monitored the offspring over the course of 12 weeks, and conducted various behavioral assays on the offspring at 12 weeks old in order to identify developmental and neurodevelopmental deficits in infected mice compared with mock-infected mice. The E16.5 time point was chosen in this and our previous study (26), as it equates to the late second trimester of human gestation and gestational weeks 20–26 of human neurodevelopment, which is the peak of neurogenesis and astrogliogenesis (59). Using this time point ensured that a variety of neural cells were present in the brain and, thus, able to be infected. We evaluated these mice using several behavioral tasks known to measure anxiety-like and memory-related behaviors, such as the OF, NOL, and NOR tests, and corroborated the results by evaluating cellular and architectural changes in the neural tissue.

Our results show that both male and female mice infected in utero with SARS-CoV-2 displayed developmental deficits such as slowed growth and lower weight in adulthood and also showed behavioral deficits such as increased anxiety-like behavior and locomotion changes. Interestingly, infected females had increased locomotion and hyperactivity, while males had decreased locomotion and a higher tendency to display freezing behavior, which was measured using the OF and Y-maze tests. Anxiety-like behavior primarily stems from the amygdala region of the brain; thus, we evaluated the morphology of the amygdala in our infected and mock-infected mice. We found that the infected mice had significantly increased amygdala volume compared with the mock-infected mice, and previous studies have shown this increased volume to be highly correlated with increased anxiety (60–62).

We then conducted the NOL and NOR tests and found that both sexes displayed significantly decreased discrimination during the NOL test compared with the mock-infected mice, but interestingly, only females displayed a decreased discrimination during the NOR test. The NOL test is used to assess aberrant hippocampal function by examining hippocampal-dependent spatial memory and, therefore, decreases discrimination indicate dysfunction of this brain region (63, 64). Importantly, both of these tests are considered a reliable and translatable means to model behavioral deficits that occur in humans using rodent models. Furthermore, we observed dysregulation in neuronal integration in the dentate gyrus of the hippocampus in both male and female infected mice by measuring the angle at which newborn neurons project into the dentate gyrus. These mice showed differences in growth angle up to 30°, and this has also been reported in the hippocampus of epileptic rodents (65). These neurons are integrated through radial glial support to provide proper hippocampal-dependent functioning; thus, alterations may lead to hippocampal-dependent behavioral deficits, such as those seen in this study.

To further evaluate the mechanisms of dysfunction, we evaluated morphological changes in the hippocampus of infected mice and found a decrease in volume in the infected males but an increase in volume in infected females. While NOL relies primarily on hippocampal function, NOR relies on various regions of the brain, such as the cortex, amygdala, and hippocampus (63, 66). We found no difference in cortical thickness in infected compared with mock-infected mice, but the changes in amygdala and hippocampal volume, as well as the dependence of this task on a more complex neural network, may provide a mechanism for the decreased discrimination in females. This reliance on a complex network may also explain the differential behavioral effect of increased NOR discrimination seen in infected males. Furthermore, various exposures during fetal brain development can result in sex-dependent alterations in brain activity and connectivity, including maternal proinflammatory cytokines (67). Although we found undetectable levels of maternal inflammation at 48 hpi in our previous study and did not measure maternal cytokine levels here (26), it is possible that even low levels of maternal cytokine exposure may result in neurodevelopmental alterations, potentially offering an additional explanation for the sex-dependent differences seen here.

Since our previous study found that in utero exposure resulted in increased levels of gliosis in the neonatal brains at 7 dpi, we investigated whether this persisted into adulthood. Imaging analysis of adult brains at 14 weeks did not show increased levels of GFAP or Iba1, which are indicators of astrocytic and microglial activation, respectively. However, we also measured the expression of TREM2 in these brains, since it is a risk factor for neurodegeneration and microglial dysfunction, and did find a slight decrease in expression in prenatally infected males, with a significant decrease in infected females. As a repressor of inflammatory cytokine production, TREM2 deficiency is associated with increases in unregulated neurotoxic inflammation (55), which could lead to cognitive and behavioral dysfunction.

In addition, we investigated whether seropositivity to the SARS-CoV-2 NP persisted after 14 weeks and determined whether the level of seropositivity correlated to the degree of behavioral change as a result of an initial stronger immune response. We found no correlation between these 2 variables; however, we did see that persisting seropositivity was observed primarily in females rather than males. This is consistent with previous literature, as it is well-established that females have greater antibody responses than males following infection and immunization, having elevated humoral immunity and maintaining higher viral titers much longer (68, 69).

Finally, in severe cases of COVID-19, or in patients with high risk of mortality, a combination drug of nirmatrelvir and ritonavir, Paxlovid, is prescribed to treat the viral infection. Since it is also prescribed to pregnant women with COVID-19 (70), we investigated whether Paxlovid treatment would treat maternal infection and prevent vertical transmission to the fetus. In our study, we found that nirmatrelvir and ritonavir eliminated SARS-CoV-2 infection in 50% of pregnant dams, with the other 50% maintaining the same levels of infection as the untreated control group. The interpretation of these results has limitations, however, as the lack of efficacy in 50% of the mice could be due to the higher metabolism in mice, which may have required a higher dosage of each antiviral. Other studies have shown efficient elimination of virus at higher dosages (71), but the dosage used in our study was chosen for proof of concept and was equivalent to the clinically used dosage in patients. Another reason for the lower efficacy could be that a longer treatment duration was needed, as other studies have found higher elimination of virus after 5 days of treatment (72), whereas we only treated until the dams gave birth (2 days of treatment).

Altogether, our study has important implications regarding the consequences of maternal COVID-19 infection in pregnant women and the effect of this infection on offspring neurodevelopment and cognition later in life. Although global agencies have declared an end to the COVID-19 pandemic and mortality rates have largely dropped, there have been over 770,000,000 cases globally since early 2020 (73). It is unknown how many pregnant women have been infected over the course of the pandemic, but understanding the potential effects that may arise in children who were exposed in utero and experienced direct CNS infection can help to guide interventions at an early stage in order to mitigate developmental and behavioral deficits.

This study is not without limitations, however. Although our mouse model replicates the effects seen in pregnant women, there may be discrepancies in the severity of the developmental and neurodevelopmental effects seen in humans due to metabolic and cognitive differences between the species. Furthermore, it has been reported that the hACE2-KI mouse model used here may have a higher level of ACE2 expression in the brain than is expressed in the human brain (74). Additionally, conducting our studies in an ABSL3 limited the assays that could be performed; therefore, our understanding of the behavioral effects is limited. We also used the Delta variant of SARS-CoV-2 in this study, so further investigation into the effects of other variants is necessary.

## Methods

### Sex as a biological variable

Mice of both sexes were used to account for sex as a biological variable.

### Study design

The overall objective of this study was to investigate the behavioral phenotypes associated with prenatal COVID-19 exposure in adult offspring, and the cellular consequences on neurodevelopment and neural circuits following prenatal COVID-19 exposure. Groups of mice were infected with defined concentrations of SARS-CoV-2, and cohort 2 was treated with defined dosages of antivirals nirmatrelvir and ritonavir. The infections and treatments included mock infections or vehicle treatments. Sample size determination was performed on the basis of prior work (26, 75), and the number of animals per group is indicated in the figure legends. Statistically significant outliers were removed from data for analysis. Animals were randomly assigned to groups by the experimenter, and all experiments were conducted in a blinded fashion. All experiments included 3 technical replicates and at least 3 biological replicates.

### Mouse strains

Eight-week-old specific-pathogen–free male and female B6.129S2(Cg)-Ace2<tm1(ACE2)Dwnt>/J (stock no. 035000) hACE2-KI (76) and J:ARC(S) (stock no. 34608) JAX Swiss Outbred mice were purchased from The Jackson Laboratory and were bred to produce the colony.

### Prenatal mouse infections with SARS-CoV-2

For prenatal infection studies, 8- to 12-week-old hACE2-KI females were bred using timed breeding, and pregnant mice were then transferred to the ABSL3 at E16.5 and housed in microisolator cages with sterile water and chow ad libitum. Mice were randomly assigned to groups and immediately infected intranasally (i.n.) at E16.5 with mock (PBS) or with 1 × 10^5^ FFU of Delta variant SARS-CoV-2, isolate hCoV-19/USA/PHC658/2021 (Lineage B.1.617.2) (BEI Resources), in a final volume of 60 μL following isoflurane sedation. After viral infection, mice were monitored daily for morbidity (body weight loss). Immediately following birth, all pups from both treatment groups were cross-fostered to JAX Swiss Outbred mice to account for aberrant maternal care in infected groups. All pups were weighed weekly on the same day for 12 weeks.

### Behavioral testing of mice

All behavioral tests were conducted inside a biosafety cabinet in the ABSL3 due to SARS-CoV-2 infection. Due to this, no photo or video recording of experiments was allowed and all timing was conducted manually. For all experiments, the experimenter was blinded to the identity of the group while scoring the experiment. Behavioral experiments were conducted on all mice at 12 weeks of age.

#### OFT.

Mice from each group were analyzed for anxiety-like behavior using the OFT. The OF arena consisted of an open acrylic box measuring 31 × 31 × 31 cm and a grid floor with square zones of equal sizes divided into a central zone and a peripheral zone. Mice were allowed to freely roam in the arena for 10 minutes, and the time spent in each zone along with the time spent moving and freezing was measured in seconds. After each session, the arena and grid flooring were sanitized with 70% ethanol to remove olfactory cues and organic waste from the mice.

#### NOL.

The NOL arena consisted of an open, light gray acrylic box measuring 31 × 31 × 31 cm with red lines on one wall as a visual cue and a grid floor with square zones of equal sizes. NOL testing consisted of 3 phases: habituation, sampling, and testing. Mice were first habituated to the empty arena for 10 minutes 1 day prior to testing. On testing day, the mice were habituated to the biosafety cabinet in their home cage for 30 minutes. The sampling phase was then conducted by placing each mouse individually in the arena with 2 sample objects for 5 minutes. Mice were then returned to their home cage for a 5-minute delay, and 1 object was moved to an adjacent corner of the cage. For the testing phase, mice were returned to the arena for 3 minutes and exploration of each object was recorded in seconds. Exploration was defined as active interaction of the mouse with the object, such as climbing on, whisking, or sniffing of the object from a distance of > 2 cm. After each session, the arena and grid flooring were sanitized with 70% ethanol to remove olfactory cues and organic waste from the mice. Discrimination index was calculated by the formula:

(Moved object exploration time/total exploration time) − (familiar object exploration time/total exploration time) × 100

#### NOR.

For NOR, all procedures were identical to the NOL test with the testing phase of the NOR test consisting of the presentation of a novel object rather than one object being relocated.

#### Y-maze spontaneous alternation test.

The Y-maze used in this study was composed of 3 equally spaced arms (120°, 31 × 5 × 31 cm) labeled A, B, and C, and was made of light gray resin. Each mouse was placed in the middle of the same arm and was allowed to roam freely for 10 minutes. An arm entry consisted of all 4 paws within the arm. The sequence of arm entries was manually recorded, and the maze was sanitized after each session with 70% ethanol. An alternation was defined as consecutive entry into all 3 arms, while a triad was defined as total number of entries minus 2. Percent alternation was calculated by the formula: (number of alternations)/(number of triads) × 100.

### Sample collection and processing

To confirm infection in dams, dams were euthanized immediately following birth, and the lungs were collected and homogenized in TRIzol Reagent (Invitrogen) for qPCR. After completion of behavioral experiments in adult offspring at 14 weeks of age, cheek blood for serum was harvested from all mice for ELISA analysis. For histological analysis, mice were perfused with PBS and 4% PFA, and then the brains were harvested and placed in 10% neutral buffered formalin for 7 days in order to inactivate SARS-CoV-2. For genomic RNA qPCR analyses, tissues were homogenized in 2 mL of PBS; then, tissue homogenates were centrifuged at 3,214*g* for 15 minutes, and supernatants were collected and placed in TRIzol. For drug treatment experiments, pups were harvested at P1.5 and brains were collected and processed for genomic RNA qPCR as described above.

### RNA isolation and qPCR analysis for quantification of SARS-CoV-2 genome equivalents (GE) per mL

Virus-infected tissues were inactivated by mixing homogenized samples with TRIzol Reagent (Invitrogen) in clean screw-top microcentrifuge tubes. Viral RNAs were extracted from the inactivated samples using an EpMotion M5073c Liquid Handler (Eppendorf) and the NucleoMag Pathogen kit (Macherey-Nagel). Briefly, 10 μg yeast tRNA and 1 × 10^3^ pfu of MS2 phage were added to each sample. After centrifugation (12,000*g*), the aqueous phase was transferred to a new tube containing NucleoMag B-Beads and binding buffer, and the samples were mixed for 10 minutes at room temperature. RNA extraction was completed on the liquid handler according to the NucleoMag Pathogen kit protocol. The isolated RNAs were quantified using a NanoDrop One spectrophotometer (Thermo Fisher Scientific). qPCR was performed on a QuantStudio 3 instrument (Applied Biosystems) using the TaqPath 1-Step RT-qPCR Master Mix, CG (Thermo Fisher Scientific) and the following cycling parameters: hold stage (2 minutes at 25°C, 15 minutes at 50°C, 2 minutes at 95°C) and PCR stage (45 cycles of 3 seconds at 95°C and 30 seconds at 60°C). The CDC-developed 2019-nCoV_N1 assay was used to measure the quantity of GE. Standard curve method was used for GE number calculations. The cut-off for positivity (limit of detection [LOD]) was established at 10 GE per reaction (800 GE/mL). Samples were tested in duplicate.

### ELISA

Seropositivity to the SARS-CoV-2 NP from serum harvested from mock and infected mice was measured by ELISA using commercial kits specific for mouse Nucleocapsid Protein IgG Antibody (ABclonal, RK04178) according to the manufacturer’s protocols. Briefly, samples were mixed with a diluent and were aliquoted in duplicate into microtiter strip wells coated with respective biotin-conjugated antibodies. Antibodies labeled with streptavidin-horseradish peroxidase were then added to the wells and incubated for 1 hour at room temperature. After the incubation, wells were washed 3 times before the addition of enzyme substrate. Samples were then incubated for 30 minutes. The resulting yellow acid dye at 450 nm with a reference filter of 630 nanometers was measured by a plate reader.

### Histology and confocal microscopy

Mouse brain samples were fixed for 7 days in 10% neutral buffered formalin and were then transferred to 30% sucrose solution at 4°C for 5 days. The tissues were then embedded in OCT Compound (Thermo Fisher Scientific) and sectioned at 16 μm on a standard cryostat using Fisherbrand Superfrost Plus Microscope Slides to collect sections. For IHC, sections were blocked and permeabilized for 1 hour at room temperature using 0.3% Triton and 3% donkey serum buffer, then incubated overnight at 4°C with the following primary antibodies: human anti–SARS-CoV-2 spike protein conjugated with Alexa Fluor 647 (1:200, Thermo Fisher Scientific, 51-6490-82), chicken anti-MAP2 (1:2,000, Thermo Fisher Scientific, PA1-10005), rabbit anti-Iba1 (1:1000, Wako Chemicals, HNM3505), rat anti-GFAP (1:2000, Thermo Fisher Scientific, 13-0300), and rabbit anti-DCX (1:200, Thermo Fisher Scientific, MA5-32495). DAPI was added following secondary incubation at room temperature for 10 minutes in the dark with the following secondary antibodies at 1:1,000: Alexa Fluor 488 (Thermo Fisher Scientific, A32790 [rabbit]; A21208 [rat]) and Cy3 (Jackson ImmunoResearch, 703-165-155 [chicken]). Slides were washed in TBS in between each incubation. Images from the brain were acquired using a Zeiss LSM 710 Confocal Microscope equipped with 4 laser lines (405, 488, 561, and 633 nm) under 20× and 40× objective lenses and Zen 2011 software. Stitching during acquisition was performed to image whole structures. Serial *Z*-stack images were collapsed to obtain a maximum intensity projection of lines after acquisition. Compared samples were processed in parallel, and the same settings and laser power were used for confocal microscopy.

IHC analysis was performed using ImageJ (NIH) and graphed using GraphPad Prism 9. DCX angle was calculated using the angle tool on ImageJ. Primary dendrite angle was calculated by comparing the angle of the primary dendrite of a cell body to the subgranular zone. Data were plotted as angle variance from 90°, as DCX dendrites properly integrate perpendicular to the subgranular zone.

Nissl staining was performed on separate sections prepared in the same manner as those used for IHC. Nissl was performed as previously described (77). Volumetric analysis of the hippocampus and amygdala was conducted by measuring the area of these regions in ImageJ (NIH) using the drawing tool. Cortical thickness was measured by determining the distance from the top and bottom of the cortical layer of the brain. Three sections per mouse were averaged, and 3 mice per group were analyzed.

### Drug treatment experiments

Pregnant mice from cohort 2 were randomly assigned to treatment groups (infected and treated), and infected i.n. at E16.5 with mock (PBS) or with 1 × 10^5^ FFU of Delta variant SARS-CoV-2. Mice were then treated twice a day for 2 days via oral gavage starting 24 hpi (Figure 1A). Dosages consisted of 5 mg of nirmatrelvir (Thermo Fisher Scientific, 502260114) and 1.5 mg ritonavir (Thermo Fisher Scientific, 461222500), comparable with the FDA-approved dosages of 300 mg of nirmatrelvir and 100 mg ritonavir in humans > 40 kg, in simple syrup. While the current treatment regimen in humans consists of 5 days of treatment, mice in this study were treated for 2 days due to peak infection at 48 hpi, determined from our previous study (26), and birth at 3 dpi. Immediately following birth, lung and brain tissues were harvested from dams of all treatment groups for genomic RNA qPCR analysis. Pups from all treatment groups were weighed, and then brains were harvested from all pups for genomic RNA qPCR analysis.

### Statistics

SigmaPlot power analysis of previous work was used to determine sample size for each experiment (78, 79). All data are reported as mean ± SEM unless otherwise indicated. Differences were considered statistically significant when *P* < 0.05. In all cases, the stated *n* value represents individual mice. For IHC, compared samples were processed in parallel, and the same settings and laser power were used for confocal microscopy. Outliers were defined by performing Grubb’s test. Two-tailed Student *t* tests and repeated-measures 1-way ANOVA were used to evaluate statistical significance, using Bartlett’s test to verify sphericity in multivariate analyses. All statistical analyses were conducted using GraphPad Prism software (version 9.3.1).

### Study approval

All experimental procedures were conducted following *Guide for the Care and Use of Laboratory Animals* (National Academies Press, 2011) and were approved by the IACUC at the University of Texas at San Antonio. All experiments using live animals or noninactivated tissues were performed inside of a biosafety cabinet in the ABSL3 at UTSA and adapted to conditions established by the Federal Select Agent Program. 

### Data availability

The data that support the findings of this study are available upon request from the corresponding author. Values for all data points in graphs are reported in the Supporting Data Values file.

## Author contributions

CLM and JH contributed to conceptualization, visualization, project administration, methodology, investigation, funding acquisition, and writing, review, and editing of the manuscript drafts. EMH and AMP contributed to methodology, investigation, and review and editing of the manuscript drafts. JH supervised all aspects of the project. ME contributed to investigation. DJL contributed to conceptualization and methodology.

## Supplementary Material

Supplemental data

Supporting data values

## Version 1 05/23/2024

In-Press Preview

## Version 2 07/08/2024

Electronic publication
