# Supplementary material for: Prenatal SARS-CoV-2 infection results in neurodevelopmental and behavioral outcomes in mice
Source: JCI Insight. 2024 May 23;9(13):e179068. doi: 10.1172/jci.insight.179068 (PMC11383367; doi:10.1172/jci.insight.179068)
Supplement: Supplemental data [file jciinsight-9-179068-s172.pdf]

Supplementary Materials for

Prenatal SARS-CoV-2 infection results in neurodevelopmental and behavioral outcomes in mice

McMahon *et al.*

Corresponding author: Jenny Hsieh, jenny.hsieh@utsa.edu

This PDF file includes:

Figs. S1 – S9

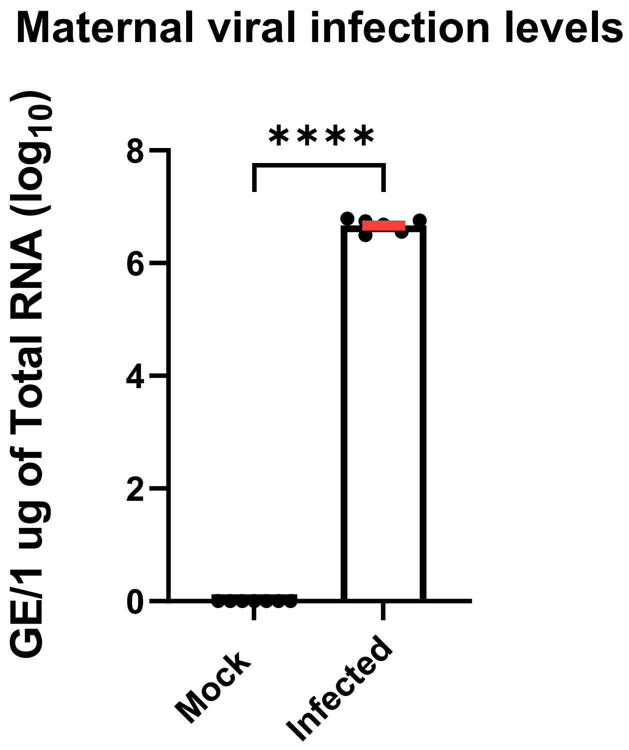

Figure S1. Confirmation of maternal SARS-CoV-2 infection. Lung tissue of mock-infected and infected dams was harvested immediately following birth for RT-qPCR analysis. n = 6

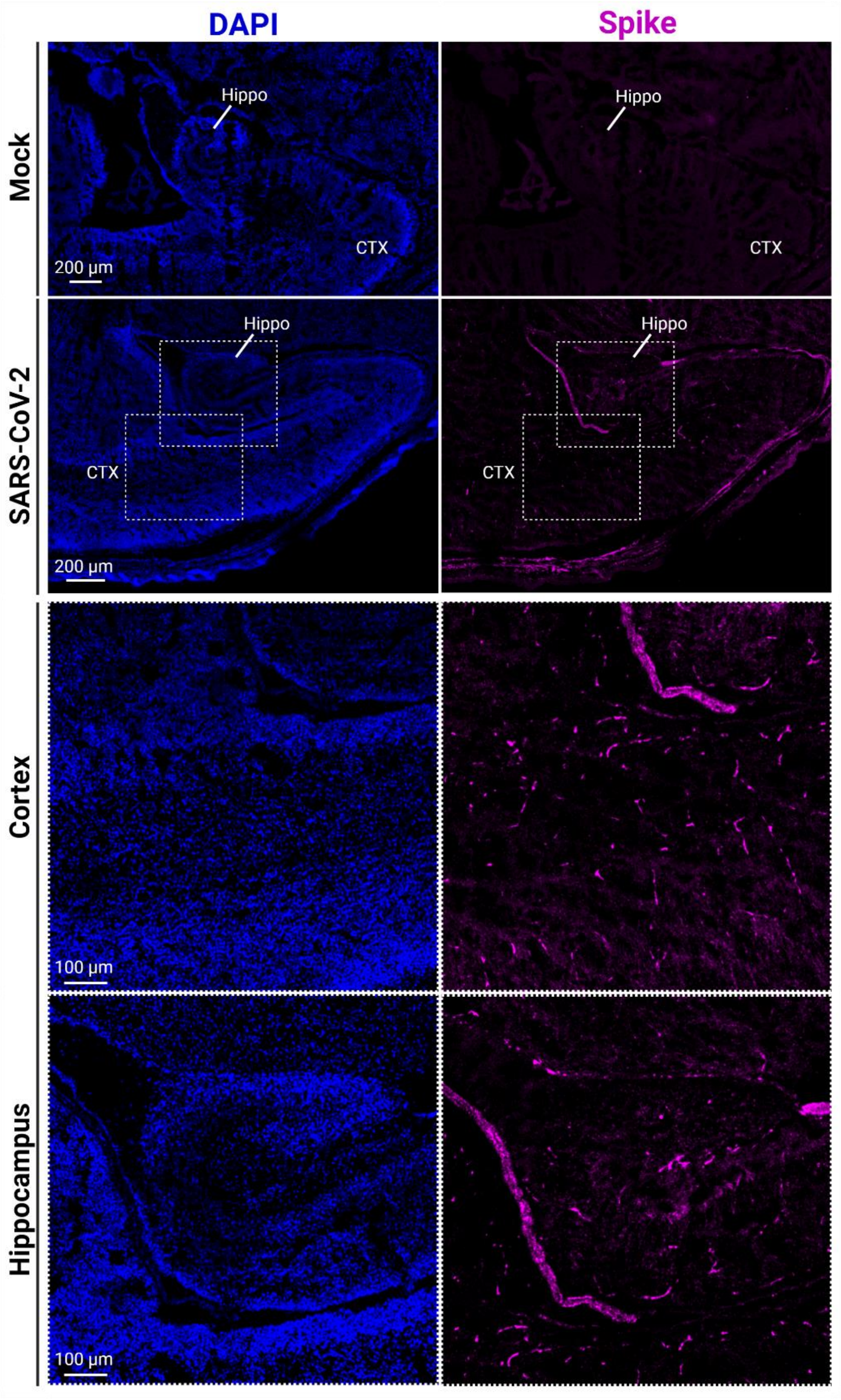

Figure S2. SARS-CoV-2 infection in the cortex and hippocampus of P1 brains. n = 6

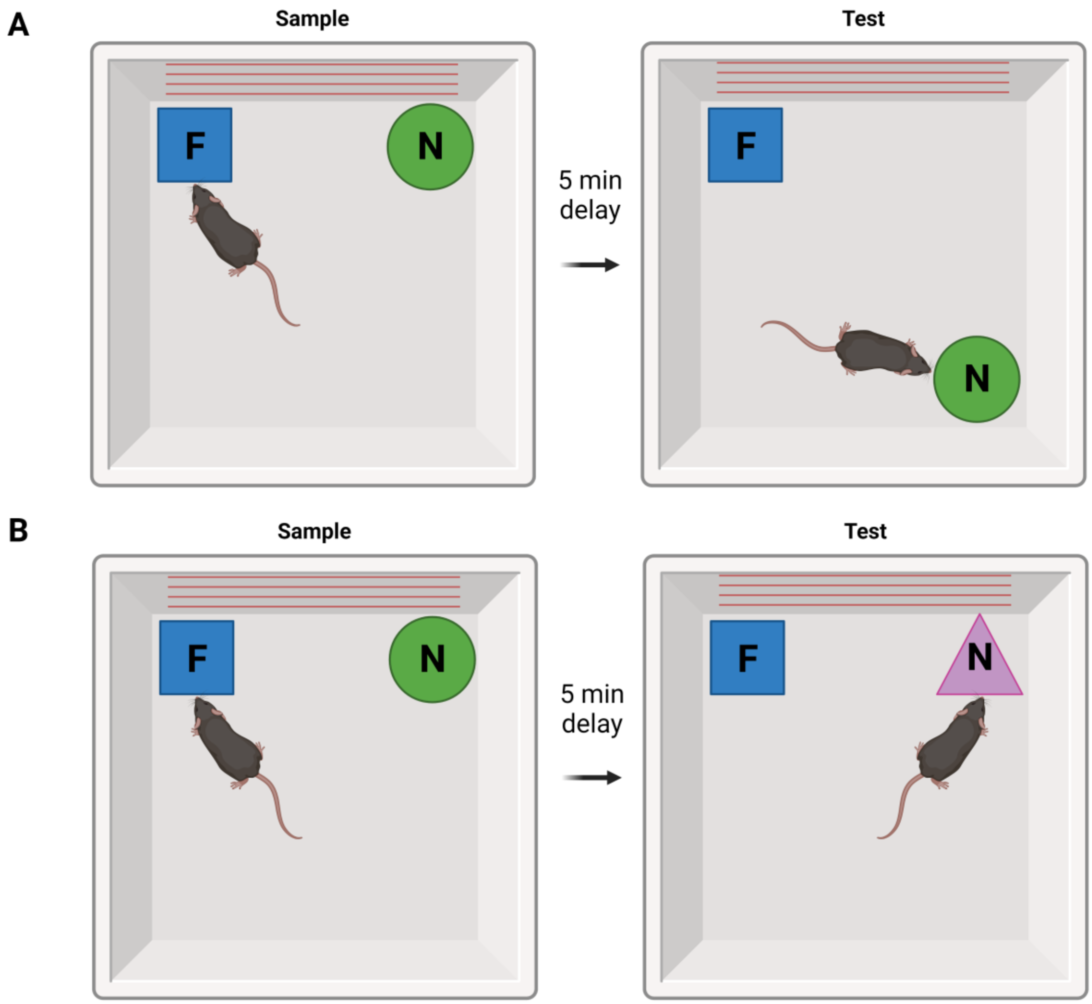

Figure S3. NOL and NOR procedure. (A) Schematic of NOL test. (B) Schematic of NOR test.

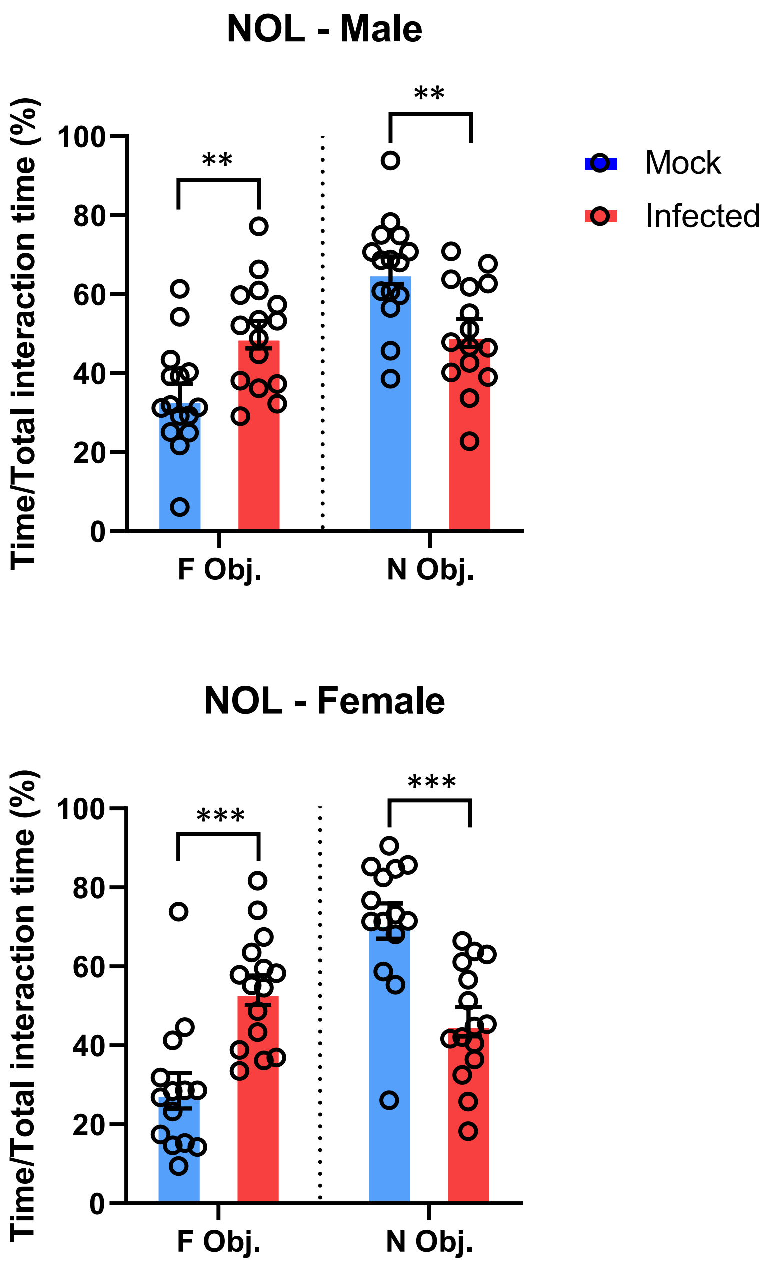

Figure S4. Time spent interacting with novel object in NOL test. Significant outliers removed for analysis. n = 14–15

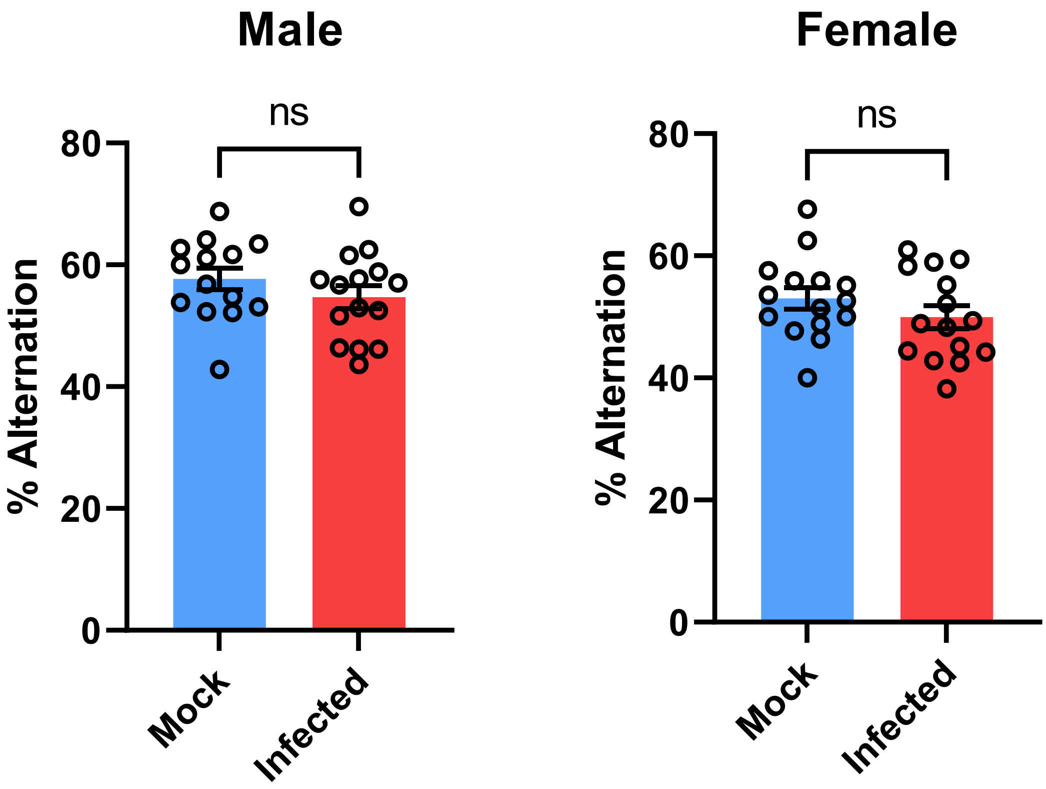

Figure S5. Percent alternation from Y-maze spontaneous alternation test. Significant outliers removed for analysis. n = 14–15

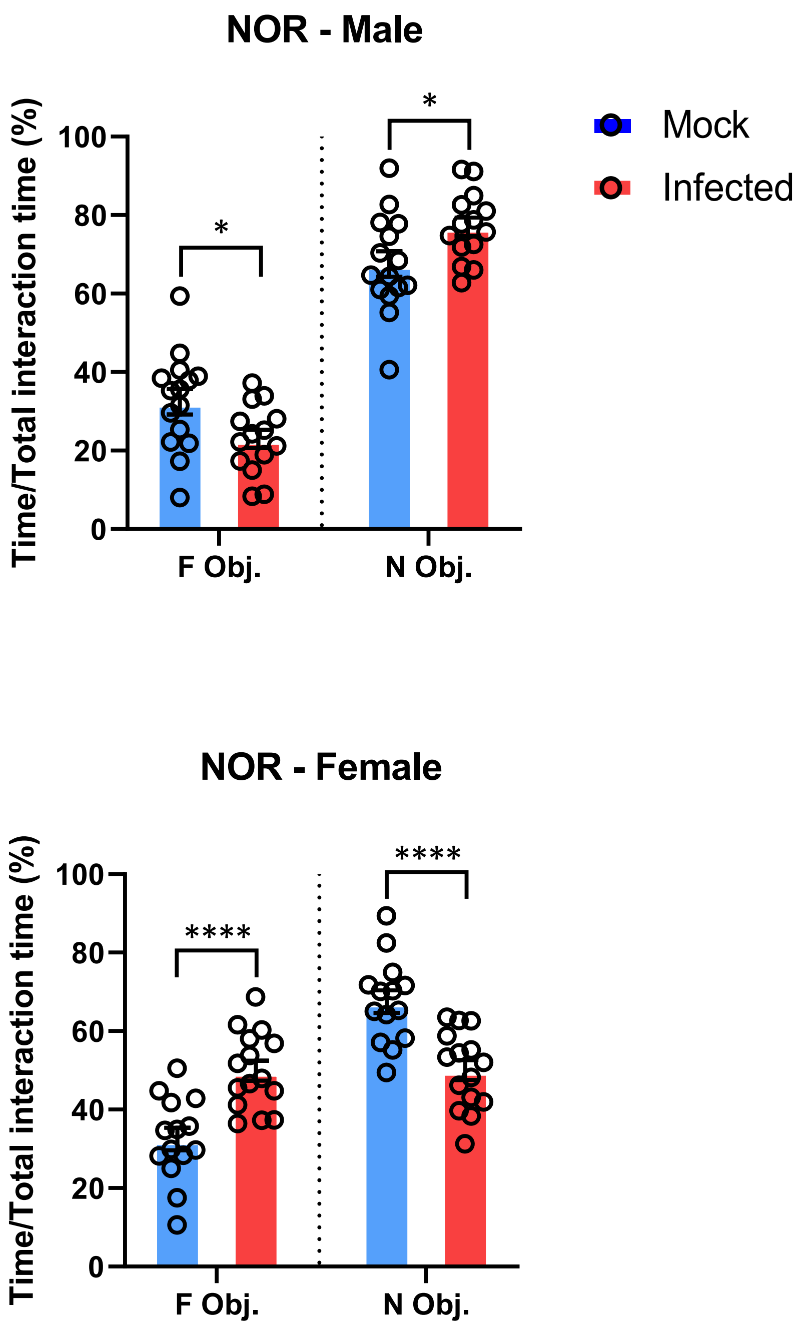

Figure S6. Time spent interacting with novel object in NOR test. Significant outliers removed for analysis. n = 14–15

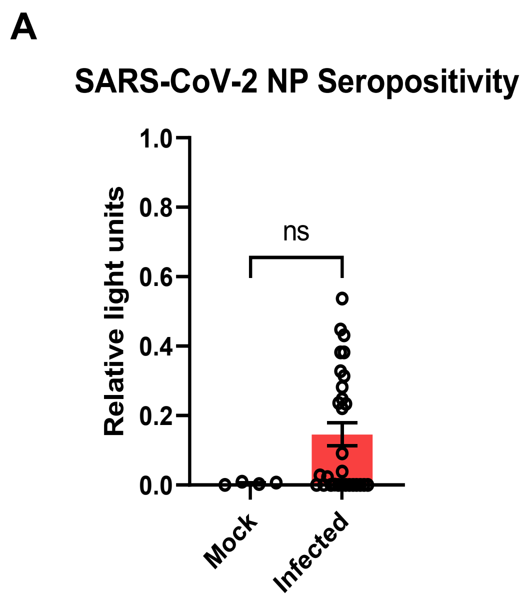

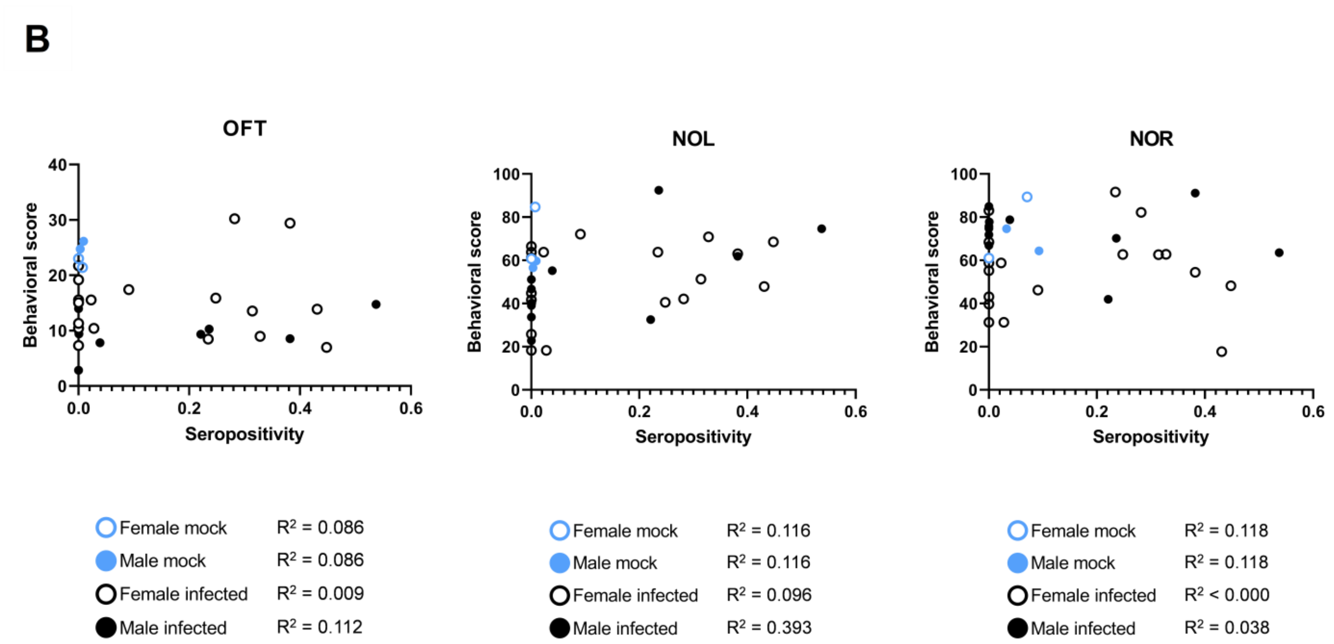

Figure S7. Seropositivity to SARS-CoV-2 NP persist at 14 weeks, but is not correlated with behavioral phenotype. (A) ELISA of seropositivity to NP. n = 4 mock/30 infected. (B) Behavioral score versus seropositivity in mice.

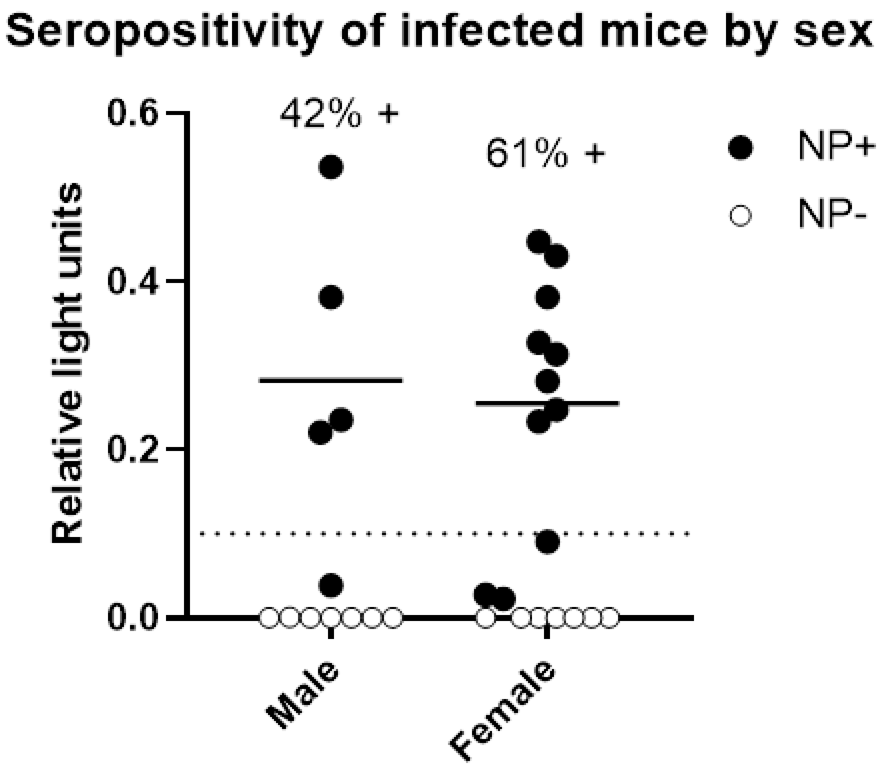

Figure S8. Seropositivity of infected mice by sex. Significant outliers removed for analysis.
n = 5–11

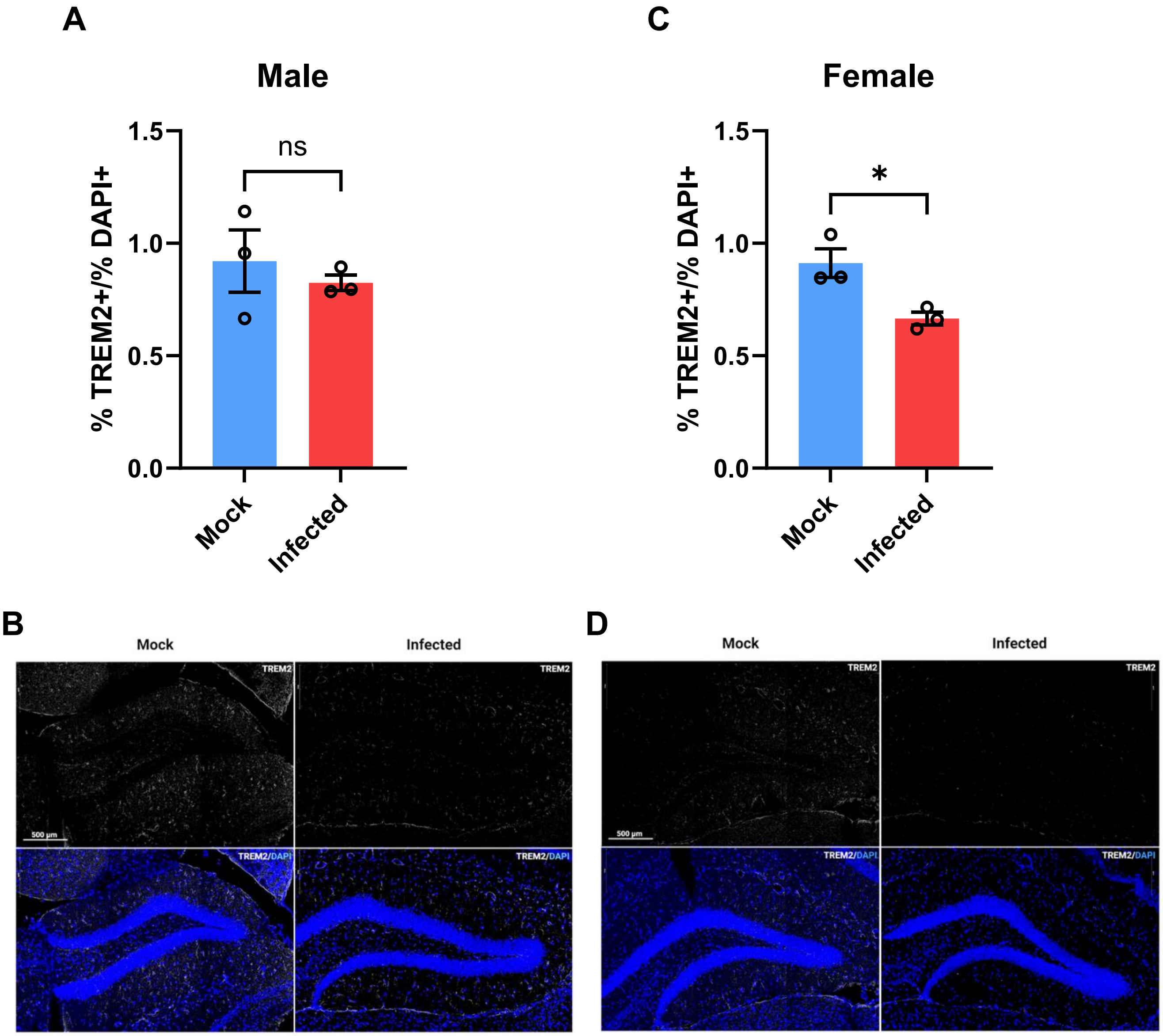

Figure S9. Percent TREM2 fluorescence expression. TREM2 expression was measured through immunohistochemistry and is not decreased in male (A-B), but is in female (C-D), infected mice compared to mock- infected mice. n = 3
